# Supplementary material for: Histone H3K36me2-Specific Methyltransferase ASH1L Promotes MLL-AF9-Induced Leukemogenesis
Source: Front Oncol. 2021 Oct 8;11:754093. doi: 10.3389/fonc.2021.754093 (PMC8534482; doi:10.3389/fonc.2021.754093)
Supplement: Supplementary file 1 [file DataSheet_1.pdf]

.

Supplementary Information

Histone H3K36me2-specific methyltransferase ASH1L promotes the MLL-AF9-induced leukemogenesis

Mohammad B. Aljazi, Yuen Gao, Yan Wu, George I Mias, Jin He
Correspondence to: hejin1@msu.edu

This PDF file includes:

Supplementary Table 1 to 3

Supplementary Table 1.Result of gene ontology enrichment analysis of genes upregulated in the MLL-AF9-tranformed cells.

Term	Count	P-Value	FDR
GO:0002376~immune system process	64	5.89E-19	1.94E-15
GO:0045087~innate immune response	57	9.25E-14	1.52E-10
GO:0006954~inflammatory response	46	2.60E-10	2.85E-07
GO:0006897~endocytosis	28	6.75E-08	5.55E-05
GO:0001974~blood vessel remodeling	12	2.76E-06	0.001812751
GO:0002224~toll-like receptor signaling pathway	8	5.57E-06	0.003049695
GO:0001774~microglial cell activation	8	8.74E-06	0.004107539
GO:0070269~pyroptosis	6	4.26E-05	0.017495751
GO:0002755~MyD88-dependent toll-like receptor signaling pathway	7	5.05E-05	0.018451926
GO:0050707~regulation of cytokine secretion	6	7.51E-05	0.024686387
GO:0043277~apoptotic cell clearance	7	1.08E-04	0.0318869
GO:0001525~angiogenesis	26	1.16E-04	0.0318869
GO:0016042~lipid catabolic process	16	1.40E-04	0.035497258
GO:0048704~embryonic skeletal system morphogenesis	11	1.81E-04	0.042510487

Supplementary Table 2. Result of gene ontology enrichment analysis of genes down-regulated in the MLL-AF9-tranformed cells.

Term	Count	P-Value	FDR
GO:0006955~immune response	52	5.56E-14	2.19E-10
GO:0006954~inflammatory response	50	4.13E-09	8.14E-06
GO:0000188~inactivation of MAPK activity	10	2.47E-08	3.25E-05
GO:0045766~positive regulation of angiogenesis	25	8.71E-08	6.78E-05
GO:0032496~response to lipopolysaccharide	33	9.88E-08	6.78E-05
GO:0007155~cell adhesion	59	1.03E-07	6.78E-05
GO:0002376~immune system process	50	1.32E-07	7.45E-05
GO:0006935~chemotaxis	23	9.09E-07	4.48E-04
GO:0030593~neutrophil chemotaxis	17	1.40E-06	6.12E-04
GO:0008285~negative regulation of cell proliferation	47	2.04E-06	8.04E-04
GO:0045785~positive regulation of cell adhesion	15	2.43E-06	8.72E-04
GO:0010628~positive regulation of gene expression	47	5.76E-06	0.001894
GO:0042102~positive regulation of T cell proliferation	15	1.30E-05	0.003949
GO:0050900~leukocyte migration	11	1.42E-05	0.004009
GO:0018108~peptidyl-tyrosine phosphorylation	15	1.57E-05	0.004133
GO:0006915~apoptotic process	59	1.82E-05	0.004475
GO:0001525~angiogenesis	32	1.99E-05	0.004609
GO:0070374~positive regulation of ERK1 and ERK2 cascade	27	3.01E-05	0.006592
GO:0042127~regulation of cell proliferation	30	4.82E-05	0.010003
GO:0050731~positive regulation of peptidyl-tyrosine phosphorylation	18	6.08E-05	0.011981
GO:0042493~response to drug	39	6.78E-05	0.012734
GO:0000122~negative regulation of transcription from RNA polymerase II	68	1.04E-04	0.017976
GO:0097192~extrinsic apoptotic signaling pathway in absence of ligand	11	1.05E-04	0.017976
GO:0043065~positive regulation of apoptotic process	38	1.14E-04	0.018508
GO:0007204~positive regulation of cytosolic calcium ion concentration	22	1.17E-04	0.018508
GO:0002687~positive regulation of leukocyte migration	8	1.64E-04	0.023961
GO:0043306~positive regulation of mast cell degranulation	7	1.75E-04	0.024678
GO:0007169~transmembrane receptor protein tyrosine kinase signaling	17	1.83E-04	0.024914
GO:0009612~response to mechanical stimulus	13	2.19E-04	0.028781
GO:0014068~positive regulation of phosphatidylinositol 3-kinase signaling	13	2.56E-04	0.032509
GO:0006334~nucleosome assembly	17	2.91E-04	0.03588
GO:0048711~positive regulation of astrocyte differentiation	6	3.55E-04	0.042358
GO:0001569~patterning of blood vessels	10	3.66E-04	0.04242
GO:0001938~positive regulation of endothelial cell proliferation	13	3.98E-04	0.044845
GO:0050728~negative regulation of inflammatory response	15	4.28E-04	0.046884
GO:0038083~peptidyl-tyrosine autophosphorylation	10	4.45E-04	0.04742

Supplementary Table 3. Primers used in this study.

Name	Sequence (5'-3')	Purpose
mHoxa9_F1	CGC CGG CAA CTT ATT AGG TG	ChIP
mHoxa9_R1	CCG ACC CGC CGA AAT TAT GA	ChIP
mHoxa9_F2	CTA CGC TCC AGG GAC CCT	ChIP
mHoxa9_R2	CTG GAA GCT GCA AGG ACT GA	ChIP
mHoxa9_F3	GTA TAT GCG CTC CTG GCT GG	ChIP
mHoxa9_R3	GCG GTT CAG GTT TAA TGC CA	ChIP
mHoxa9_F4	AGG ACC GAG CAA AAG ACG AG	ChIP
mHoxa9_R4	GGG AGA GGA GAC AGA GGG AG	ChIP
mHoxa10_F1	CTG CTA CAG GGC CCG TTT AA	ChIP
mHoxa10_R1	TTA TGA TGT GCA CCC CAG CC	ChIP
mHoxa10_F2	GCG TCT TCT GGC CCA TCA AT	ChIP
mHoxa10_R2	CGA CCA CTC CCA GTT TGG TT	ChIP
mHoxa10_F3	TCT GCT CCC TTC GCC AAA TT	ChIP
mHoxa10_R3	CCT CTG CCT GAG CTG ATG AG	ChIP
mHoxa10_F4	GGA GTG CTG GGC TGT GTT TA	ChIP
mHoxa10_R4	CTC TGC TCT TGG CCA AGG AA	ChIP
mAsh1_1F	CCCACACAAATGTAAGTTTGGA	Genotype
mAsh1_1R	ACATGGAGTTATTAGATCCTG	Genotype
mAsh1_2F	AGC CTG ACT GGC CTA GAA TG	Genotype
mAsh1_2F	TCC AAA CTG TAG AGC AGA AAA CA	Genotype
